# Advancing Equity for Family Caregivers: Rural-Urban Differences in Demands, Resources, and Outcomes

**DOI:** 10.1093/geroni/igaf122.1000

**Published:** 2025-12-31

**Authors:** Everlyne Ogugu, Orly Tonkikh, Jessica Famula, Heather Young, Janice Bell, Bronwyn Fields

**Affiliations:** Betty Irene Moore School Of Nursing at UC Davis, Sacramento, California, United States; University of Haifa, Haifa, HaZafon, Israel; University of California, Davis, Davis, California, United States; Betty Irene Moore School Of Nursing at UC Davis, Sacramento, California, United States; Betty Irene Moore School Of Nursing at UC Davis, Sacramento, California, United States; Sacramento State University, Sacramento, California, United States

## Abstract

In 2020, 55.8 million US adults were ≥65 years old, making up 16.8% of the US population, and this number is expected to rise to 80.8 million by 2040. With increases in the proportion of older adults, the demand for caregiving increases, particularly in rural communities with a higher proportion of older adults. An examination of rural versus urban caregiver needs, and resource availability is a crucial initial step toward providing effective and equitable support services for caregivers. This study examined caregiver experiences, caregiving intensity, caregiver resources (availability of paid or unpaid help, satisfaction with social support) and caregiver health outcomes (self-rated health, depressive symptoms) for rural-dwelling caregivers in comparison with suburban/urban-dwelling caregivers. We used assessment survey data of caregivers from the California Caregiver Resource Centers (CCRC) online platform, CareNav (n = 5,782). We used logistic regression models to assess association between place of residence and caregiving intensity, caregiver resources and health outcomes and negative binomial models to explore the association of place of residence and count outcomes (caregiving tasks, memory, and behavioral problems). The proportion of rural dwelling caregivers was 4.4% and 95% of caregivers identified as the primary caregiver. Rural dwelling caregivers reported managing 9% more memory and behavior problems whereas urban dwelling caregivers were 25% more likely to report experiencing difficulties in managing medical-surgical nursing tasks. There were no statistically significant differences between rural and suburban/urban-dwelling caregivers in caregiving intensity, caregiver resources or health outcomes. This study highlights differences in caregiving demands with implications for program delivery.

